# Why Not Glycine Electrochemical Biosensors?

**DOI:** 10.3390/s20144049

**Published:** 2020-07-21

**Authors:** Clara Pérez-Ràfols, Yujie Liu, Qianyu Wang, María Cuartero, Gastón A. Crespo

**Affiliations:** Department of Chemistry, School of Engineering Science in Chemistry, Biotechnology and Health, KTH Royal Institute of Technology, Teknikringen 30, SE-100 44 Stockholm, Sweden; clarapr@kth.se (C.P.-R.); yujiel@kth.se (Y.L.); qianyuw@kth.se (Q.W.); mariacb@kth.se (M.C.)

**Keywords:** glycine, electrochemical sensors, point-of-care, healthcare, biosensing

## Abstract

Glycine monitoring is gaining importance as a biomarker in clinical analysis due to its involvement in multiple physiological functions, which results in glycine being one of the most analyzed biomolecules for diagnostics. This growing demand requires faster and more reliable, while affordable, analytical methods that can replace the current gold standard for glycine detection, which is based on sample extraction with subsequent use of liquid chromatography or fluorometric kits for its quantification in centralized laboratories. This work discusses electrochemical sensors and biosensors as an alternative option, focusing on their potential application for glycine determination in blood, urine, and cerebrospinal fluid, the three most widely used matrices for glycine analysis with clinical meaning. For electrochemical sensors, voltammetry/amperometry is the preferred readout (10 of the 13 papers collected in this review) and metal-based redox mediator modification is the predominant approach for electrode fabrication (11 of the 13 papers). However, none of the reported electrochemical sensors fulfill the requirements for direct analysis of biological fluids, most of them lacking appropriate selectivity, linear range of response, and/or capability of measuring at physiological conditions. Enhanced selectivity has been recently reported using biosensors (with an enzyme element in the electrode design), although this is still a very incipient approach. Currently, despite the benefits of electrochemistry, only optical biosensors have been successfully reported for glycine detection and, from all the inspected works, it is clear that bioengineering efforts will play a key role in the embellishment of selectivity and storage stability of the sensing element in the sensor.

## 1. Introduction

Amino acids (AAs) play a key role in regulating the whole-body metabolism, which is essential for human health, growth, development, and survival [1]. Among all the AAs, glycine is the smallest one, having only a single hydrogen atom as its side chain. However, glycine is an important AA that accounts for ca. 11.5% of total AAs and ca. 20% of AA nitrogen in body proteins [2]. Thus, there is a significant association between glycine and many human disease states [3], which justifies glycine being one of the most attractive analytes for clinical applications. Conversely, glycine was traditionally considered a non-essential AA because it can be endogenously biosynthesized within the human body, primarily in the liver and kidneys [4]. This theory was well proved through isotopic studies that confirmed that glycine turnover occurs during body metabolism, resulting in glycine converting into different compounds (i.e., serine, urine, glutamine, alanine, and other AAs) and flowing to different body parts [5,6,7].

**Figure 1:**
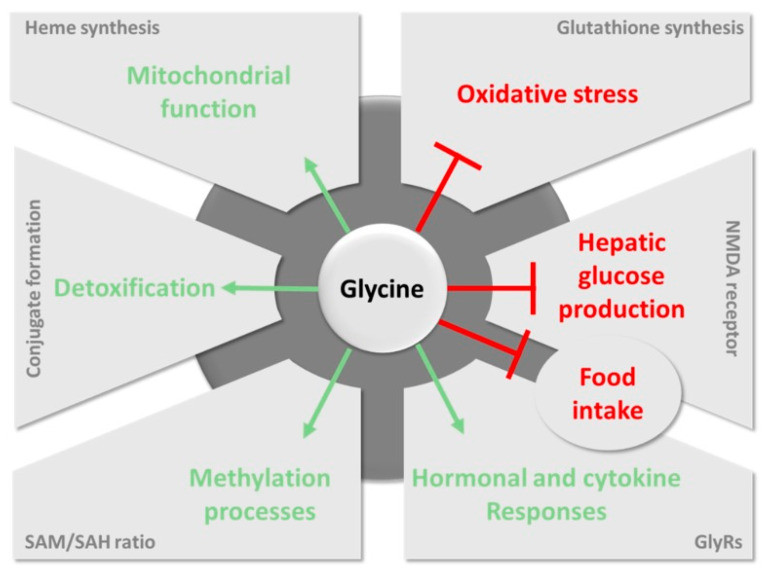
Main pathways involving glycine in health benefits. Favorable pathways induced by glycine and harmful pathways inhibited by glycine are represented in green and red, respectively. NMDA: *N*-methyl-d-aspartate; GlyRs: glycine receptors; SAM: S-adenosylmethionine; SAH: S-adenosylhomocysteine. Reproduced from ref. [15].

On the other hand, the amount of glycine endogenously synthetized is not enough to support body activity and, therefore, modern AA classifications usually term glycine as a conditionally essential (or semi-essential) AA [4,8]. From a nutritional perspective, insufficiency in dietary glycine intake is not detrimental, but a chronic inadequacy may cause severe effects on the functioning of body organism, including suboptimal growth, impaired immune responses, and other adverse effects on health and nutrient metabolism [9,10]. Additionally, numerous studies have demonstrated that dietary supplementation of glycine can improve outcomes in many clinical conditions, because it may help to prevent the infiltration of inflammatory cells [11], inhibit tumor growth [12,13], and decrease the toxicity of certain drugs [14], among others. The main health benefits mediated by glycine are illustrated in Figure 1.

As the smallest and the only non-chiral AA, glycine presents a unique structure that is irreplaceable in the framework construction of many structural proteins (e.g., collagen fibril and elastin [16]) and common metabolites (e.g., glutathione, creatine, porphyrins, purines, heme, and serine [3]) as well as in the provision of the flexibility necessary for conformational changes in the active sites of some particular enzymes [17]. Consequently, glycine is involved in multiple physiological processes that are related to three crucial functions: cytoprotection, anti-inflammatory responses, as well as body growth and development [3].

Many glycine-related disorders are associated with its synthesis and catabolism, meaning that any change that occurs in either its generation or consumption processes may induce severe syndromes in the individual. Up to now, efforts have been directed to establish an association between glycine levels and disease states, with previous research confirming that low plasma glycine levels can be related to obesity [18], diabetes [18], decreasing sleep quality [19], gout [20], and schizophrenia [21], whereas high levels of glycine result in nonketotic hyperglycinemia (NKH, also known as glycine encephalopathy) [22]. Therefore, considering the wide diversity of glycine functions and their involvement in many different organism (essential) pathways, it is not surprising that glycine is one of the most analyzed biomolecules for clinical purposes.

**Figure 2:**
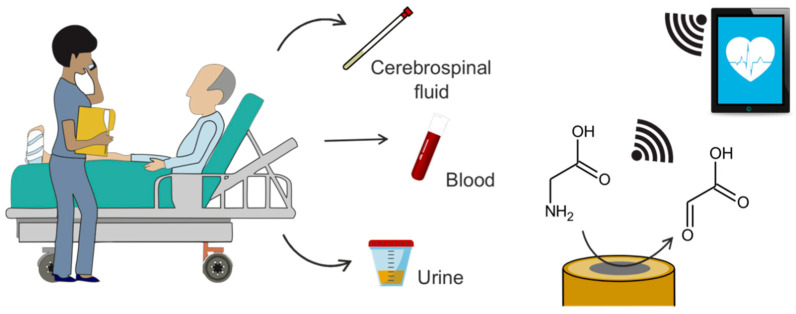
Graphic concept of electrochemical glycine determination in the clinical field. Glycine is analyzed in CSF, blood or urine through its electrochemical oxidation and the obtained data are transferred wirelessly to a phone/tablet.

The current gold standard analytical approach for glycine detection in the clinical field involves sample extraction (usually blood and/or urine) and chromatographic or fluorometric analyses in centralized laboratories, which results in long delays in results provision as well as considerable economical costs. Consequently, there is a clear need for the development of reliable point-of-care (POC) glycine detection methods able to provide real-time information related to a patient’s health status. In this context, this review focuses on the electrochemical detection of glycine as a strategy that can easily be implemented in POC devices, specifically considering biosensors (Figure 2). The first section of this review focuses on the different body fluids where glycine determination is clinically relevant, reviewing glycine levels and the information about health disorders that can be acquired from each targeted body fluid. Subsequently, the different electrochemical strategies reported up to now for glycine determination are critically discussed, with particular emphasis on their suitability for glycine quantification at the POC level. Finally, electrochemical biosensing is discussed as a potential alternative that can help toward overcoming some of the analytical drawbacks that are currently impeding the establishment of POC glycine platforms.

## 2. Main Sources of Clinically Relevant Information

The involvement of glycine in several inter-organ metabolic paths results in its presence in several biological fluids at different concentration levels. Table 1 summarizes healthy and unhealthy levels of glycine expected in different biological fluids, as per reported analyses in the clinical field. Notably, only blood (including plasma and serum), urine, and cerebrospinal fluid (CSF) are commonly investigated in routine clinical applications corresponding to glycine-related diseases [23,24,25]. Then, because glycine levels in all the biological fluids are in very similar ranges, the same analytical technique should, in principle, be able to cover the analysis of any fluid. However, this is not the case, as far as we know, as a consequence of presenting different matrix effects that impede this kind of universality.

Quantitative analysis of glycine in all the biological fluids has been demonstrated to provide valuable information for the diagnoses of specific diseases and the subsequent monitoring of patients’ rehabilitation [24]. For example, the early identification of NKH solely relies on the detection of glycine levels in plasma and CSF [26]. On the other hand, saliva, sweat, and interstitial fluid (ISF) are also attractive sources of biomarkers that are appealing due to the possibility of being accessed in a non-invasive way [27]. Yet, these fluids have not been routinized for glycine detection and there is rare accessible clinical information regarding human glycine levels. In addition, blood-derived fluids (plasma and serum) are normally employed instead of whole blood [28]. While these two matrices seem to demonstrate similar characteristics, their AA levels are not identical because of different pre-treatment procedures [29]. Several studies have pointed out that higher and more variable content of AAs is present in serum than in plasma and, therefore, current clinical glycine analysis generally prefers the use of plasma over serum [30] to avoid possible interferences.

As a fluid excreted from human body after regular metabolism, urine is considered another helpful matrix that gives diagnostic information [31]. Its use could indeed be considered superior compared to blood because it is non-invasive, simple, and without requirement of a specially trained operator. Besides that, urine samples could be abundantly supplied (the average value for adult is 1.5–2.0 L per day) [31]. Along with elevated plasma glycine, an excessive amount of glycine in urine is also typical among NKH patients. Mixed effect from renal function and drug treatment results in more variable glycine concentrations in urine, increasing the complexity of data interpretation compared to plasma [32]. As a result, urine glycine analysis is not generally considered as the primary preference to collect information for screening an inborn disorder of metabolism [33]. Nevertheless, in some specific applications, it is crucial to perform such detection [33]. For example, iminoglycinuria is characterized by elevated glycine levels in urine (along with proline and hydroxyproline), while plasma glycine remains at a normal level [34]. In conclusion, urinary glycine detection is still of obvious clinical significance, being used as complementary information to blood-related tests, but also as a unique diagnosis of some specific diseases.

**Table 1:** Physiological ranges of glycine concentrations (μM) in different body fluids.

Sample	Healthy Levels ^a^	Unhealthy Levels ^a^	Ref.
Plasma/Blood ^b^	147–299 (men) 100–384 (women)	450–2363	[35,36]
CSF ^c^	3.8–10	<3 and 30–1927	[22,26,35,37]
Urine ^d^	44–300 ^g^	550–5000	[31,38]
Saliva	177.80 ± 143.20	-	[39]
Sweat ^e^	1751 ± 150 (passive) 997–595 (exercise)	-	[40,41]
ISF ^f^	565 ± 92 (adipose) 400 ± 48 (muscle)	-	[42]

^a^ Literature values differentiate from laboratories and techniques. ^b^ Glycine levels in plasma depend on sex and age. ^c^ CSF glycine level also depends on age but with much less differences compared with plasma. ^d^ Urinary glycine is commonly normalized by creatinine concentration. ^e^ Sweat glycine levels vary significantly from passive sweat and sweat after different durations of exercises. ^f^ ISF concentration depends on the type of tissue considered. ^g^ μM glycine/mM creatinine.

Compared with other commonly analyzed biological fluids, CSF is harder to access [37] because its collection requires a lumbar puncture. However, glycine detection in CSF is highly valuable in the diagnosis of several disorders, among which the most noteworthy is NKH [43]. The confirmation is normally established by elevated CSF and plasma glycine levels in combination with a CSF-to-plasma glycine ratio greater than 0.08 (normal ≤ 0.02) [22,44]. The simultaneous collection (within 2 h) of CSF and plasma is necessary to provide diagnostic information [33,45]: in the collection of CSF, some precaution should be taken to avoid blood contamination, because this will generate erroneous elevation of glycine level in the CSF sample, given the much higher blood glycine range, leading to an invalid result [24].

Overall, blood plasma analysis is currently the preferred assay for clinical glycine detection [25], because it is informative, relatively easy to access, and reproducible [46]. However, it should be highlighted that diagnosis of glycine-related diseases is not recommended through the analysis of a unique biological fluid. Therefore, considering that the combination of different matrix detections is of great significance in clinical applications, an ideal glycine sensing platform should be compatible with a variety of biological fluids.

## 3. Current Analytical Methodologies for the Determination of Glycine

Clinical quantitative determination of glycine in biological fluids is frequently accomplished by means of liquid chromatography methods [47,48,49]. Among them, the most common technique is ion-exchange chromatography (IEC) combined with post-column ninhydrin derivatization [45,49,50,51,52,53,54], which is able to convert glycine into a relatively stable chromophore with absorbance at λ_max_ of 570 nm (Ruhemann’s purple easily detected by ultraviolet spectrophotometry) [45,55]. With the advantages of high reproducibility and accuracy [54,56], good linearity over a wide range of concentration [24], sensitivity in the picomole level [24], and complete automation [57], this methodology was considered as the gold standard for clinical analysis of glycine (as well as other physiological AAs) over recent decades [24,44,53]. Notably, some aspects should be critically addressed, aiming at evaluating the real suitability of this approach:

- Chromatographic methods with optical detection seem to lack specificity towards glycine determination [24,47]. As separation and identification are exclusively based on the retention time, there is always a risk of AA coelution [24,45], which could lead to overestimated results. For example, co-elution of glycine, arginine, histidine, and valine is reported for HPLC when the ionic strength of the eluent is not properly chosen [56]. Essentially, a careful selection of the stationary and mobile phases is mandatory to provide appropriate results, which is evidently more expensive, as a particular combination is exclusively used for only one analyte.
- Sample pre-treatments are complicated while inevitable, namely deproteinization and derivatization [47,58,59]. The former process is necessary because of the presence of soluble interferent species, such as peptides and proteins in the fluid, that will encumber the chromatographic column and give elevated backpressure in the instrument [60]. Accordingly, these compounds will disturb both quantitative and qualitative analysis, in addition to generating negative impacts on the instruments. Then, it is essential to include derivatization processes in column-based methods with optical detection because neither chromophore nor fluorophore groups are present in the molecular structure of glycine [47,61]. However, this treatment will bring negative effects to the analysis as well, as a consequence of the presence of derivatized compounds (impurities) [47].
- A typical IEC AA analysis normally takes several hours due to the fact that a low mobile phase flow rate is needed (for example 0.25 mL/min [62,63]) [58], which is quite time-consuming [54,59,64]. Furthermore, there is always extra time spent on mobile phase elution between each measurement for the purposes of removing residual impurities from the previous sample as well as equilibrating the column before the analysis of a new sample. This long analysis results in an important delay between the extraction of the sample and the outcomes’ provision, and therefore, the implementation of any needed medical treatment.
- The instrumentation and maintenance of the IEC instrument (and therefore, the related analyses) are costly [64]. Chromatographic methods require specialized equipment [48] that small-sized hospitals and laboratories might not have access to. Furthermore, these techniques demand for skillful operators that should be capable of implementing testing on the exquisite facilities [59]. The combination of these two aspects implies that glycine analysis is mainly performed in specific centralized laboratories. Thus, after collecting the sample, transportation to external laboratories is many times indispensable, resulting in an even extended overall time of test and data provision [65].

As alternatives to the conventional IEC method, other chromatographic systems have been proposed in routine glycine analysis [60,66], such as reversed phase high-performance liquid chromatography (RP-HPLC) with pre-column derivatization [67] and ultra-high-performance liquid chromatography (UPLC) [68]. Although these approaches have shown shorter operation time (45 min is reported for UPLC) [68] and higher sensitivities [56], this type of method still displays common weaknesses related to chromatographic systems, which have just been discussed.

Another well-established tool for glycine detection in biological fluids is a commercial fluorometric kit that can be acquired from many different providers [69,70,71,72,73,74]. These fluorometric kits are based on an enzymatic assay in which glycine is oxidized, yielding a fluorometric product. Notably, to utilize the intrinsic linear range of calibration offered by the kit, different degrees of dilution are required for the analysis of serum, urine, and saliva, attending to the different amounts of glycine (see Table 1). In addition, further dilutions as well as deproteinization of samples may be required to avoid interferences from proteins and common metabolites present in biological fluids. Apart from time consumption, this sample pre-treatment is likely to affect the analysis results by introducing several systematic errors [27]. Moreover, specific sample volume is necessary to generate strong enough fluorescence signal, which naturally correlates to a relatively large consumption of reagents in the kit components. High price and relatively long operation time (sample incubation solely takes 1 h) make this technology not suitable for extensive application.

With those considerations in mind, modern glycine sensing is currently in demand of the establishment of a rapid, direct, robust, and simple methodology that can be adapted to POC platforms. Compared with conventional chromatographic methods, electrochemical sensors depict unique advantages, such as rapidity, miniaturized size, low cost, and less energy consumption [75,76,77]. Thereby, this sensing methodology is now attracting more attention and many efforts have been devoted towards new glycine (bio)sensors. In this direction, the next sections critically review all the reported electrochemical glycine sensors, pointing out future development possibilities in the field.

### Electrochemical Sensors for the Determination of Glycine

**Figure 3:**
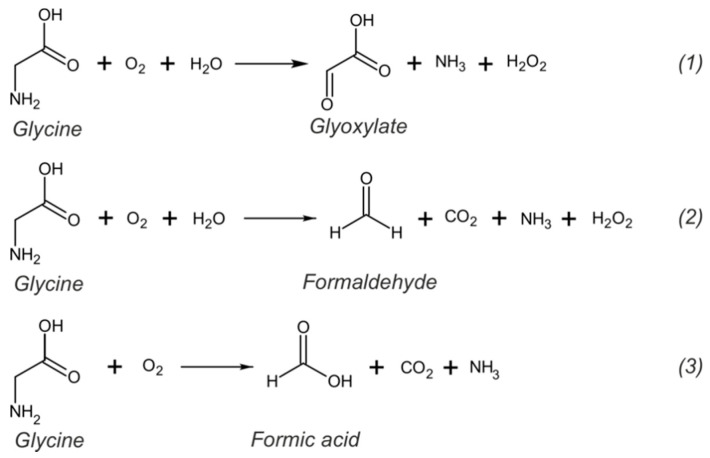
Possible routes for oxidation of glycine.

Electrochemical sensors for the determination of glycine are usually based on glycine oxidation. As an aliphatic AA, in the presence of oxygen, glycine can undergo mainly three different oxidation reactions, as shown in Figure 3, resulting in the formation of glyoxylate, formaldehyde or formic acid [78]. All these reactions require high oxidation potentials and are not favored at bare carbon electrodes because of slow electron transfer rate [77,79]. Indeed, very low signals (and even no signal) with low sensitivity and reproducibility are usually observed at unmodified carbon electrodes. Therefore, the electrochemical determination of glycine requires an electrocatalyst or redox mediator to assist in lowering the oxidation potential and increase the electron transfer rate.

**Figure 4:**
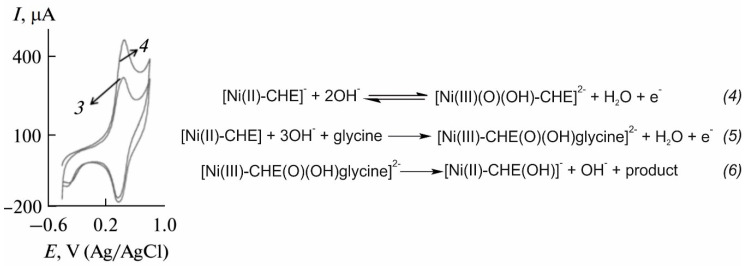
Reactions involved in the determination of glycine using a Ni(II) chelidamic acid-modified electrode with the expected cyclic voltammograms in the absence (*reaction 4*, curve 3) and presence (*reactions 5 and 6*, curve 4) of glycine. (The plot for the voltammograms on the left is reproduced from reference [61] with permission from Springer, Copyright 2020).

The first group of electrocatalysts reported to provide suitable results in the electrochemical determination of glycine was based on metal complexes. As observed in Table 2, some of the metal complexes applied to the determination of glycine include Ni chelidamic acid [61], Ni(II)–baicalein complex [80], Fe(III)–Schiff base [79], Ni(II) hydroxide [81] and cobalt hydroxide oxide [82]. In this type of electrode, metal complexes act as redox mediators and a change in their oxidation signal is recorded. As an example, the mechanism of the electrocatalytic oxidation of glycine at the Ni chelidamic acid-modified electrode is shown in Figure 4. In the absence of glycine, Ni chelidamic acid is oxidized to a Ni(III) oxohydride complex (*reaction 4*), resulting in an oxidation peak at ca. 0.4 V. In the presence of glycine, glycine is adsorbed onto the surface and oxidizes Ni(II) centers to Ni(III) (*reaction 5*). Finally, Ni(III) complex oxidizes glycine, being simultaneously reduced to Ni(II) chelidamic acid (*reaction 5*) and giving rise to an increased intensity of the anodic peak, which is proportional to the glycine concentration in the sample solution. Analogous mechanisms can be observed for the rest of the metal complexes reported.

This strategy can be implemented with many amperometric techniques, with chronoamperometry and differential pulse voltammetry (DPV) being the two most frequent. In terms of analytical performance, metal complex-based electrodes can provide limits of detection (LOD) in the range of a few µM and linear ranges of response from a few µM up to approximately 1 mM, even reaching 12 mM in the case of the Fe(III)–Schiff base-modified electrode [79]. These parameters are, in principle, suitable for the direct determination of glycine on the most common biological fluids, because glycine levels are usually at the µM range (see Table 1). However, only the nickel chelidamic acid-modified electrode was actually applied to the determination of glycine in human serum and, even in this case, the samples had to be diluted (1.5:2 in methanol) and the proteins extracted to minimize interferences [61]. Furthermore, one of the reasons that prevents the direct determination of glycine in undiluted biological fluids using these electrodes is that the optimal response of metal complex-based electrodes is commonly achieved at extreme pH values, either 2 or 13, which differs significantly from the physiological pH. Therefore, some sample pre-treatment is always needed with this type of electrodes.

A possible explanation to the lack of applications using the mentioned electrodes in either diluted or undiluted real samples may be the low selectivity provided by metal complex-based electrodes. Taking into account the working mechanism previously explained, an increased metal complex signal is to be observed not only in the presence of glycine but also in the presence of any other species which oxidation can be catalyzed by the metal complex. This depends on the type of interaction established between the metal complex and glycine compared to potential interferences: these electrodes are usually also responsive to other AAs with similar structure to glycine (e.g., serine, alanine or valine). Furthermore, the high potentials required for the oxidation of glycine could also allow the oxidation of other common electroactive species. For example, the Ni(II)–baicalein complex showed important response towards glucose, ascorbic acid, uric acid, citric acid, and urea [80]. As pointed out in some reports, this multi-response could be interesting in combination with flow systems or separation techniques (such as chromatography or electrophoresis) with the advantage of avoiding the tedious derivatization procedures usually required in optical detectors [83,84]. Evidently, this low selectivity is a serious drawback in pursuing the direct determination of glycine, which is crucial for the development of POC platforms. This is indeed one of the fields in which electrochemical sensors can make a remarkable difference.

An improved selectivity was achieved with a paraffin-impregnated graphite electrode modified with ruthenium hexacyanoferrate (RuHCF)/reduced graphene oxide [75]. In this work, the synergy of reduced graphene oxide with RuHCF provided an improved electrocatalytical performance, being capable of discriminating between glycine, glutathione, and threonine (i.e., separate peaks for each AA through DPV). In addition, no interference was observed between the three analytes or from ascorbic acid, uric acid, dopamine, l-cysteine, aspartic acid, salicylic acid, tartaric acid, or urea. This electrode was successfully applied to the simultaneous determination of glycine, glutathione, and threonine in a spiked human saliva sample. However, the matrix had to be diluted 100 times and the pH adjusted to 5 in order to obtain accurate results, as a result of a very narrow linear range of response (from 1.25 to 7.49 μM), which is indeed too low for the direct analysis of glycine in any undiluted body fluid except for CSF.

Other voltammetric and amperometric sensors reported for the determination of glycine are based on metal nanoparticles [86,87], silica mobile crystalline materials [86,88] or biopolymers [77]. Among these, the NiO NPs-modified rotating glassy carbon electrode presented a behavior similar to metal complex-based electrodes, with an optimized response at pH 13 and similar LOD, although the linear range of response was narrower (from 1 to 200 μM) and the higher concentration too low for measuring undiluted samples. Apart from glycine, this electrode was also responsive to alanine and serine, but with neglected response for threonine, asparagine, histidine, glutamine or proline.

A deeper study regarding AAs interference was carried out by Hasanzadeh et al. [86], using a glassy carbon electrode modified with magnetic silica mobile crystalline material-41 (Si-MCM-41-Fe_2_O_3_). Oxidation peak potentials were indeed related to the AA structure, observing similar potentials for AAs containing similar functional groups. Moreover, higher currents were observed when the structure of the AA allowed a greater interaction with Si-MCM-41-Fe_2_O_3_. In particular, glycine was oxidized at 0.83 V, presenting a similar potential as tryptophan. Advantageously, the Si-MCM-41-Fe_2_O_3_-modified electrode presents an optimized response at pH 8.1, which is much closer to physiological pH than previously described electrodes. Although a much lower LOD was presented, the linear range of response was again very narrow, only reaching up to 1 μM. Importantly, a slightly lower LOD and wider linear range of response (0.1–1.2 μM), although still far from the normal levels found in biological fluids, was obtained at physiological pH by functionalizing Si-MCM-41 with amino groups [88].

Adequate responses at physiological pH with a wide linear range of response up to 70 μM were observed with a glassy carbon electrode modified with poly-dopamine-beta-cyclodextrin [77]. In this case, the electrocatalytic effect was a result from the combination of active functional groups in the surface of poly-dopamine that could easily form hydrogen bonds with the amine groups of glycine, and the presence of beta-cyclodextrin, which allowed for the inclusion of glycine in its hydrophobic cavity providing a sort of preconcentration. This was manifested as a decrease in the oxidation potential of glycine (ca. 0.14 V), which is indeed expected when chemically and orientally favoring the oxidation process.

Even though voltammetric and amperometric sensors are the most common for the electrochemical determination of glycine, some sensors based on other electroanalytical techniques have also been reported. Alam et al. [76] reported on a glassy carbon electrode modified with ZnO/Al_2_O_3_/Cr_2_O_3_ nanoparticles and interrogated by electrometry at pH 7. Thus, the metallic nanoparticles were able to electrocatalyze the oxidation of glycine to formaldehyde, giving rise to electrons’ generation that increased the conductivity of the solution. This method provided a very low LOD (82.25 pM) with a linear range of response limited to very low concentrations (up to 1 μM). An interference study showed that the presence of common ions (K^+^, Na^+^, Fe^2+^ or Ca^2+^) did not affect to the sensor response but other biomolecules (ascorbic acid, bilirubin, d-fructose, d-glucose, l-cystine, l-glutathione, tannic acid, and uric acid) strongly affected the electrode response, with no possibility to differentiate among them. This method was successfully applied to the determination of glycine in spiked human, mice, and rabbit serum samples.

A different approach was proposed by the group of B. Yaroslavtsev [64,85]. In this case, the electrochemical determination of glycine was based on Donnan potential sensors using semipermeable membranes based on cation exchanger architectures (Nafion or MF-4SC). SiO_2_ and/or ZrO_2_ nanoparticles were used as dopants, creating an electrostatic repulsion that results in the widening of the membrane pores. Thus, glycine is easily transferred from the sample solution to the membrane. Advantageously, an optimization of the membrane (material, dopant concentration, and doping strategy) allows the increase in the sensitivity towards glycine, while reducing the interference from potassium (pH > 7) or protons (pH < 7). Although no information was provided regarding linear range of response, this methodology seems to work at the mM range. The simultaneous determination of glycine, alanine, leucine, and potassium was achieved by combining different pairs of sensors that showed the highest sensitivity towards each AA and the lowest correlation among them.

## 4. Towards the Direct and Decentralized Glycine Electrochemical Detection

An ideal POC sensing platform should demonstrate the eight features established by the ASSURED guidelines [89]: Affordable, Sensitive (minimal false negatives), Specific (minimal false positives), User-friendly, Rapid, Robust, Equipment-free, and easily Delivered to end users. Currently, chromatographic methods and fluorometric kits provide high sensitivity, specificity, and robustness, whereas electrochemical sensors easily fulfil affordability, user-friendliness, and rapidness. However, as far as we know, none of the available methods for glycine determination in the clinical field are able to meet simultaneously all the ASSURED criteria.

**Figure 5:**
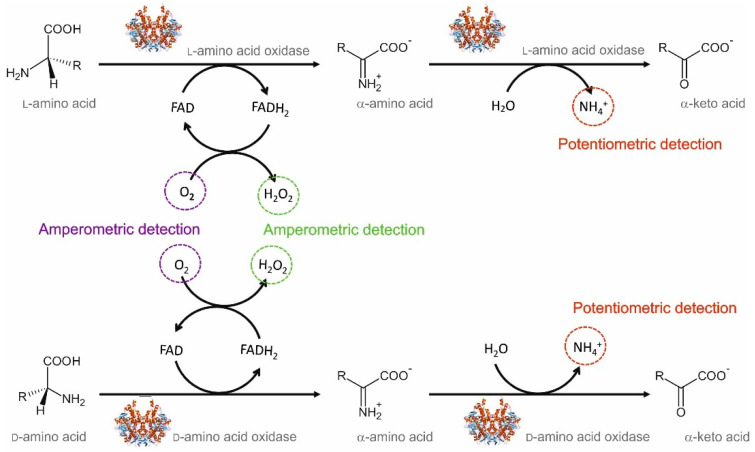
Main approaches for the biosensing determination of l-AAs (upper row) and d-AAs (lower row) based on electrochemical detection.

In the field of electrochemical sensors for glycine determination, the main challenge that needs to be addressed is selectivity, as discussed in the previous section. The integration of biosensing elements in such electrodes could be a trustable alternative. This strategy will benefit from the specificity of enzymes and may help improve the poor selectivity, as already demonstrated for other common analytes such as glucose, lactate or urea, among others. In this context, biosensors for the determination of AAs have been recently reviewed by Pundir et al. [90]. The main approaches reported for electrochemical biosensing of AAs are illustrated in Figure 5. The most common enzymes used in these biosensors are d-amino acid oxidase (DAAO, EC No. 1.4.3.3) and l-amino acid oxidase (LAAO, EC No. 1.4.3.2), which are two flavoproteins that convert d-AAs and l-AAs, respectively, into the corresponding α-keto acids and ammonium using oxygen and releasing hydrogen peroxide. In this scheme, AA determination is most frequently based on the amperometric detection of hydrogen peroxide, although sensors that monitor the consumed oxygen or the generated ammonia have also been reported [90,91,92].

Strategies based on the amperometric detection of hydrogen peroxide (Figure 5, in green) usually require the introduction of a redox mediator to decrease the high overpotential associated with the direct hydrogen peroxide determination, which would favor the interference of other electroactive species present in the sample. Common redox mediators are based on transition metal compounds, conducting polymers, and organic dyes-based mediators [93]. In addition, interferences may also be reduced by introducing a permselective membrane. For example, Lata et al. [94] developed a biosensor for total l-AA determination in fruit juices and alcoholic beverages, based on the covalent immobilization of LAAO onto a carboxylated multiwalled carbon nanotubes/nickel hexacyanoferrate/polypyrrole hybrid film electrodeposited on the surface of a glassy carbon electrode. In this biosensor configuration, the hybrid film containing carbon nanotubes, nickel hexacyanoferrate (a Prussian blue analog), and polypyrrole enhances the electron transfer between the electrode surface and the enzyme, while at the same time, providing a more biocompatible environment for the enzyme. The proposed sensor was tested for the determination of l-phenylalanine as a representative l-AA, achieving a LOD of 0.5 μM and a wide linear range of response between 0.5 μM and 100 mM. Negligible interferences were observed for ascorbic acid, acetic acid, ethanol, citric acid, cysteine, uric acid, glucose, fructose, sodium, and potassium. However, as this biosensor was proposed for the determination of total l-AA, the discrimination between different l-AA was not studied. The accuracy of this biosensor was tested through the determination of several fruit juices and alcoholic beverages, achieving a good correlation with the results provided by a standard colorimetric method.

More in the clinical field, Zain et al. [95] reported a biosensor based on DAAO that was applied to the determination of d-serine in rat brain tissue, providing a LOD of 20 nM and a response time of 0.7 s. In this case, amperometric detection of peroxide hydrogen was carried out at Pt-Ir (90/10%) by applying a potential of 0.7 V. The use of such high oxidation potential entailed the incorporation of permselective membranes to avoid interferences. For this purpose, both Nafion and poly-ortho-phenylenediamine (PPD) membranes were incorporated, which allowed the decrease in the interference from ascorbic acid (the main interferent) to barely 0.05%. In addition, no interferences were observed for common species in the central nervous system (CNS), such as l-AA, dopamine, uric acid, DOPAC, 5-hydroxyindole acetic acid or homovanillic acid. This biosensor did respond to d-alanine with higher sensitivity than for d-serine, although this interference is not too problematic for brain tissue analysis because the levels of d-alanine in CNS are about two orders of magnitude lower than those of d-serine. In vivo measurements were carried out by implanting the developed sensor in the brain of anaesthetized rats. Although the d-serine values obtained were not fully validated, the performance of the implanted biosensors was evaluated through the direct injection of 5 µL of 100 mM d-serine next to the sensor, which resulted in an increase in the current recorded.

Another strategy that allows the amperometric determination of peroxide hydrogen is based on a bienzymatic configuration, where horseradish peroxidase (HRP, EC 1.11.1.7) catalyzes the reduction in peroxide hydrogen. This scheme was reported by Domínguez et al. [91], who tested this bienzymatic configuration for both l-AA and d-AA by modifying composite graphite–Teflon electrodes with HRP and either LAAO or DAAO. The reduction in peroxide hydrogen was mediated by ferrocene. These biosensors were individually tested for different l- and d-AAs, achieving LODs in the range of 1–200 µM depending on the considered AA. In general, lower sensitivities were observed for polar AAs, which authors attributed to the hydrophobic nature of the Teflon present on the surface. Both biosensors were able to correctly discriminate enantiomers in racemic samples but were unable to distinguish between different AAs.

Although less common, amperometric biosensors for AA determination may also be based on the monitoring of the consumed oxygen (Figure 5, in purple). This strategy is usually less popular due to some drawbacks associated with oxygen monitoring: oxygen concentration is not constant in real samples and the high concentration of oxygen in aqueous solutions hinders the determination of low AA concentrations. Nevertheless, Zhang et al. [96] developed a biosensor for homocysteine determination in human plasma based on DAAO immobilized eggshell membrane and an oxygen electrode. The proposed biosensor could easily operate at physiological pH and provided a LOD and linear range of response of 30 µM and 0.05–1.5 mM, respectively. Taking into account that normal levels of homocysteine in plasma usually range from 5 to 16 µM, the analytical performance of this biosensor should be further improved before it can be considered for clinical applications. Furthermore, high interferences were observed for cysteine because it can react with homocysteine to form S–S bonds, which results in consumption of the dissolved oxygen in the solution.

Alternatively, potentiometric sensors may also be applied to the determination of AAs. In this case, AA concentration is indirectly determined by measuring the amount of ammonium ions generated (Figure 5, in red). Following this strategy, Lee et al. [92] developed a potentiometric biosensor for l-AA monitoring during yeast autolysis. The proposed biosensor consisted of a nonactin-PVC membrane, which acted as an ammonium selective membrane, and an enzymatic membrane in which LAAO was immobilized onto Nylon and cross-linked with glutaraldehyde. Both membranes were attached to a commercial ammonia electrode and held by a cellulose membrane. High responses were achieved for l-isomers of phenylalanine, leucine, tryptophane, cysteine, methionine, tyrosine, and isoleucine, whereas little to no response was observed for glycine, serine, threonine, proline, glutamate, and aspartate. Within the first group, phenylalanine and leucine showed the widest linear ranges of response (0.01–10 mM).

Because the main purpose of DAAO- and LAAO-based biosensors is to discriminate between d-AAs and l-AAs, little attention is usually placed on glycine, being the only non-chiral AA. As a result, the available literature really lacks biosensors for glycine detection. In fact, to the best of our knowledge, no electrochemical biosensors have been reported for glycine determination yet. Although in principle, all three measuring schemes illustrated in Figure 5 could be suitable for glycine determination, the main aspect that is currently hindering the development of a glycine biosensor is the lack of a stable enzyme that can selectively catalyze glycine oxidation. Therefore, research on different types of enzyme, both natural or bioengineered, and their substrate specificity towards glycine is indispensable for the further development of electrochemical biosensors for glycine determination. Consequently, some clues regarding alternative enzymes that could be employed in glycine biosensors will be next provided.

Glycine oxidase (GO, EC No. 1.4.3.19) is a more specific enzyme that has been reported to be capable for quantitative glycine detection as the basis of either optical biosensors preparation [97] or colorimetric assays [59]. GO is a flavoenzyme from *Bacillus subtills* that is composed of four identical structures, with each one containing noncovalently bound flavin adenine dinucleotide (FAD) [97,98,99,100,101]. GO catalyzes the oxidation reaction of glycine in the presence of water and oxygen following an analogous mechanism to that illustrated in Figure 5 for DAAO and LAAO. In particular, for glycine, the reaction results in the formation of glyoxylate [98,101]. Although GO is more specific toward glycine than LAAO or DAAO, GO is not only active for glycine: GO shares partial substrate specificity with various flavooxidases, including DAAO and sarcosine oxidase (SOX, EC No. 1.5.3.1), additionally catalyzing the oxidation of neutral d-AAs (e.g., d-alanine and d-proline) as well as primary and secondary amines (e.g., sarcosine and *N*-ethylglycine) [99,100,101]. Thus, several studies have focused on increasing GO specificity towards glycine by means of bioengineering. For example, Rosini et al. [97] engineered a total of 16 single point GO variants, some of them exhibiting improved kinetic parameters and/or a higher substrate specificity ratio for glycine versus sarcosine. The introduction of multiple mutations also provided an increased maximal activity on glycine. Importantly, one GO variant with high specificity towards glycine was applied to the development of a fluorometric biosensor for the determination of glycine in biological samples. For this purpose, two disposable and commercially available fluorometric cuvettes were used, the first filled with Red Nile (a dye transducer) and the second containing the bioengineered GO. The set-up was arranged so that the emission light (450 nm) passed through GO before reaching Red Nile and recording the fluorescence spectra from 500 to 700 nm. In the absence of glycine, the oxidized GO absorbs part of the emission light, quenching the original fluorescence intensity of Red Nile. When glycine is added onto the second cuvette, the FAD cofactor of GO is reduced and its absorbance decreases, resulting in an increase in the light that reaches Red Nile and, therefore, in fluorescence intensity. This methodology provided a LOD lower than 0.5 µM and was successfully applied to the determination of glycine in the U87 human glioblastoma cell line and human plasma samples, providing similar analytical performance as chromatographic methods, but in a much faster way (1 s against 60 min). However, the analysis of these biological samples required AA extraction and anaerobic conditions, which prevents this biosensor implementation as POC platforms.

Another important factor that needs to be considered in the development of biosensors is the stability, because there is always a risk of enzyme deactivation due to diverse reasons. For GO, this is indeed a particularly sensitive issue because GO cannot be stored for long term at 4 °C, which hampers its implementation in biosensing or commercial biological assays. Nevertheless, enzyme tunability can also result in an improved enzyme stability. Tatsumi et al. [59] developed a GO triple mutant that retained most of the enzymatic activity during storage for over a year at 4 °C. This engineered GO mutant was applied to the colorimetric determination of glycine, which was based on a double enzyme scheme. First, glycine was oxidized by GO generating hydrogen peroxide, which in turn, reacted with *N*-ethyl-*N*-(2-hydroxy-3-sulfopropyl)-3-methylaniline (TOOS) and 4-aminoantipyrine in the presence of HRP, resulting in the formation of a quinoneimine dye that was detected at 555 nm. This method presented a LOD and a linear range of response of 2.2 µM and 7–600 µM, respectively, and was successfully applied to the determination of glycine in human plasma. The obtained results were comparable to those provided by an automated AA analyzer based on chromatography, with the additional advantages of avoiding derivatization or deproteination pretreatments and providing a fast analysis (5 min) that can simultaneously be performed on multiple samples using a microplate reader. In terms of selectivity, this assay was also responsive to sarcosine, *N*-ethylglycine, d-alanine, and d-proline. However, some authors have claimed that plasma analysis was not affected because the concentration of these substances in plasma is less than 1% of that of glycine (alanine and proline are usually present as l-AAs), although extrapolation to other biological fluids should be taken carefully as, for example, sarcosine levels in saliva are around 10% of those of glycine [39].

Apart from enzymes, glycine biosensors may also be based on proteins. Zhang et al. [102] recently developed a fluorescent Forster resonance energy transfer (FRET) glycine sensor using Atu2422 protein from *Agrobacterium tumefaciens* as the recognition element. A rational design of this protein allowed authors to increase the specificity towards glycine, reducing the binding from l-serine and GABA that is expected from the non-mutated Atu2422 protein. For the construction of the fluorescent biosensor, the mutated protein was inserted between enhanced cyan fluorescent protein (ECFP) and Venus-fluorescent protein (Venus), a pair of donor-acceptor fluorophores commonly used in FRET sensors. Apart from glycine, small changes in the fluorescence ratio were also observed for leucine, valine, and threonine, although the binding for these three AAs was insignificant in the concentration range of 0–50 µM and at concentrations ratios AA/Gly > 10. This FRET biosensor was applied to the determination of glycine transients in acute hippocampal slices prepared from male Wistar rats, which allowed authors to test predictions about compartmentalization of glycine levels (i.e., synaptic, presynaptic and extrasynaptic) and investigate the mechanisms controlling glycine concentrations (e.g., pharmacological inhibition of glycine transporters and stimulation of collateral synapses at low and high frequencies).

## 5. Conclusions

Glycine analysis for clinical purposes is currently performed at centralized laboratories by means of time and cost consuming methodologies, mainly involving chromatography or fluorometric kits. Electrochemical sensors have been proposed aiming at more rapid and economical glycine analysis. The majority of these are amperometric sensors based on metal complexes and provide a suitable LOD for glycine analysis in biological fluids. However, to date, none of the reported glycine electrochemical sensors have been able to provide the necessary selectivity, linear range of response, and/or capability to operate at physiological conditions, essential features that are required for the implementation of electrochemical sensors as POC platforms. An interesting alternative today entirely lacking in the literature is based on electrochemical biosensors, i.e., containing one enzyme to derivatize the glycine to electrochemically measurable compounds. While only a few optical biosensors have been reported for glycine determination, using either GO or Atu2422 protein as sensing elements, we were not able to find any glycine electrochemical biosensor. A key aspect would be the enhancement of specificity, kinetic parameters, and storage stability through bioengineered enzymes and proteins in order to really put forward this alternative. In particular, the enhanced properties of GO mutations shed light on the development of electrochemical biosensors that will hopefully provide superior analytical features compared to already existing chemical sensors, while allowing simpler set-ups to be further implemented as POC platforms. This process will also require further efforts to adapt glycine sensors to miniaturized platforms containing the whole electrochemical cell (working, reference, and counter electrodes) that are able to perform glycine analysis either using really low amounts of sample (e.g., blood pricking) or based on wearable sensing approaches for a higher frequency monitoring.

## Figures and Tables

**Table 2:** Electrochemical sensors reported for the determination of glycine.

Sensing Element	Technique	Analytical Parameters	Interferences	Application	Ref.
RuHCF/rGO	SWV E_ox_ = −1.36 V pH 5	LOD = 0.4 μM LRR = 1.25–7.49 μM	Able to determine Gly, GSH and Thr simultaneously	Spiked saliva (diluted 100 times)	[75]
ZnO/Al_2_O_3_/Cr_2_O_3_ NPs	Electrometry pH 7	LOD = 82.25 pM LRR = 0.1–1000 nM	Interference from GSH and Cys.	Spiked human, mouse, and rabbit serum	[76]
Polydopamine-β-cyclodextrin	DPV E_ox_ = 0.14 V pH 7.4	LOD = 0.06 μM LRR = 0.2–70 μM	Interference from Cys, Tyr, Phe.	None	[77]
ZrO_2_ or SiO_2_ NPs	Potentiometry	LOD = 60 μM	Able to determine Gly, Ala and Leu simultaneously with electrode array	None	[85]
ZrO_2_ NPs	Potentiometry	NS	Able to determine Gly, Ala and Leu simultaneously with electrode array	None	[64]
Ni chelidamic acid	Amperometry E_ap_ = 0.35 V pH 13	LOD = 0.3 μM LRR = 1–750 μM	No interference from Leu, Ala or Glu.	Spiked human serum (diluting and extracting proteins)	[61]
MCM-41-Fe_2_O_3_ NPs	Amperometry E_ap_ = 0.6 V pH 8.1	LOD = 145 nM LRR = 0.3–1 μM	Interference from Cys, Val, Phe, Ser, Trp and Tyr	None	[86]
NiO NPs	Amperometry E_ap_ = 0.42 V pH 13	LOD = 0.9 μM LRR = 1–200 μM	Interference from Ser and Ala. No interference from Thr, Asn, His, Gln or Pro	None	[87]
MCM-41 functionalized by 3-aminopropyl	DPV E_ox_ = NS pH 7.4	LOD = 10.11 nM LRR = 0.1–1.2 μM	Interference from Cys, Val, Phe, Ser, Arg, Trp and Tyr	None	[88]
Fe(III)–Schiff base complex	DPV E_ox_ = NS pH 2	LOD = 4.11 μM LRR = 3–12200 μM	Interference from Cys, Val, Phe, Ser, Arg, Trp and Tyr	None	[79]
Ni(II)–baicalein complex	Amperometry E_ap_ = 0.55 V pH 13	LOD = 9.2 μM LRR = 20–1000 μM	Interference from Val, Ser, Trp and His	None	[80]
Co(OH)O NPs	DPV E_ox_ = NS pH 13	LOD = 10.02 μM LRR = 20–1500 μM	Interference from Val, Phe, Ser, Arg, Trp and Tyr	None	[82]
Ni(OH)_2_	Amperometry E_ap_ = 0.5 V pH 13	LOD = 30 μM LRR = 0.1–1.2 mM	Interference from Arg. No response to Glu, Leu or Ala	None	[81]

Ala: alanine; Asn: asparagine; CV: cyclic voltammetry; Cys: cysteine; DPV: differential pulse voltammetry; E_ap_: applied potential; E_ox_: oxidation peak potential; Gln: glutamine; Glu: glutamic acid; Gly: glycine; GSH: glutathione; His: histidine; Leu: leucine; LRR: linear range of response; MCM: mobile crystalline material; NP: nanoparticle; NS: not specified; Phe: phenylalanine; Pro: proline; rGO: reduced graphene oxide; RuHCF: ruthenium hexacyanoferrate; Ser: serine; SWV: square wave voltammetry; Thr: threonine; Trp: tryptophan; Tyr: tyrosine; Val: valine.

## References

[1] Wu G. (2010). Functional amino acids in growth, reproduction, and health. Adv. Nutr..

[2] Wu G.Y. (2010). Recent advances in swine amino acid nutrition. J. Anim. Sci. Biotechnol..

[3] Wang W., Wu Z., Dai Z., Yang Y., Wang J., Wu G. (2013). Glycine metabolism in animals and humans: Implications for nutrition and health. Amino Acids.

[4] Yu Y.M., Yang R.D., Matthews D.E., Wen Z.M., Burke J.F., Bier D.M., Young V.R. (1985). Quantitative aspects of glycine and alanine nitrogen metabolism in postabsorptive young men: Effects of level of nitrogen and dispensable amino acid intake. J. Nutr..

[5] Waterlow J.C., Golden M.H., Garlick P.J. (1978). Protein turnover in man measured with ^15^N: Comparison of end products and dose regimes. Am. J. Physiol. Endocrinol. Metab..

[6] Picou D., Taylor-Roberts T. (1969). The measurement of total protein synthesis and catabolism and nitrogen turnover in infants in different nutritional states and receiving different amounts of dietary protein. Clin. Sci..

[7] Matthews D.E., Conway J.M., Young V.R., Bier D.M. (1981). Glycine nitrogen metabolism in man. Metabolism.

[8] Gersovitz M., Bier D., Matthews D., Udall J., Munro H.N., Young V.R. (1980). Dynamic aspects of whole body glycine metabolism: Influence of protein intake in young adult and elderly males. Metab. Clin. Exp..

[9] Jackson A.A. (1991). The glycine story. Eur. J. Clin. Nutr..

[10] Meléndez-Hevia E., de Paz-Lugo P., Cornish-Bowden A., Cárdenas M.L. (2009). A weak link in metabolism: The metabolic capacity for glycine biosynthesis does not satisfy the need for collagen synthesis. J. Biosci..

[11] Wheeler M.D., Rose M.L., Yamashima S., Enomoto N., Seabra V., Madren J., Thurman R.G. (2000). Dietary glycine blunts lung inflammatory cell influx following acute endotoxin. Am. J. Physiol. Lung Cell. Mol. Physiol..

[12] Rose M.L., Madren J., Bunzendahl H., Thurman R.G. (1999). Dietary glycine inhibits the growth of B16 melanoma tumors in mice. Carcinogenesis.

[13] Rose M.L., Cattley R.C., Dunn C., Wong V., Li X., Thurman R.G. (1999). Dietary glycine prevents the development of liver tumors caused by the peroxisome proliferator WY-14,643. Carcinogenesis.

[14] Thurman R.G., Zhong Z., von Frankenberg M., Stachlewitz R.F., Bunzendahl H. (1997). Prevention of cyclosporne-induced nephrotoxicity with dietary glycine. Transplantation.

[15] Alves A., Bassot A., Bulteau A.L., Pirola L., Morio B. (2019). Glycine metabolism and its alterations in obesity and metabolic diseases. Nutrients.

[16] Wagermaier W., Fratzl P., Matyjaszewski K., Möller M. (2012). Collagen. Polymer Science: A Comprehensive Reference.

[17] Yan B.X., Sun Y.Q. (1997). Glycine residues provide flexibility for enzyme active sites. J. Biol. Chem..

[18] Adeva-Andany M., Souto-Adeva G., Ameneiros-Rodriguez E., Fernandez-Fernandez C., Donapetry-Garcia C., Dominguez-Montero A. (2018). Insulin resistance and glycine metabolism in humans. Amino Acids.

[19] Bannai M., Kawai N. (2012). New therapeutic strategy for amino acid medicine: Glycine improves the quality of sleep. J. Pharmacol. Sci..

[20] Mahbub M.H., Yamaguchi N., Takahashi H., Hase R., Amano H., Kobayashi-Miura M., Kanda H., Fujita Y., Yamamoto H., Yamamoto M. (2017). Alteration in plasma free amino acid levels and its association with gout. Environ. Health Prev. Med..

[21] Neeman G., Blanaru M., Bloch B., Kremer I., Ermilov M., Javitt D.C., Heresco-Levy U. (2005). Relation of plasma glycine, serine, and homocysteine levels to schizophrenia symptoms and medication type. Am. J. Psychiatry.

[22] Applegarth D.A., Toone J.R. (2001). Nonketotic hyperglycinemia (glycine encephalopathy): Laboratory diagnosis. Mol. Genet. Metab..

[23] Schmitt B., Steinmann B., Gitzelmann R., Thun-Hohenstein L., Mascher H., Dumermuth G. (1993). Nonketotic hyperglycinemia: Clinical and electrophysiologic effects of dextromethorphan, an antagonist of the NMDA receptor. Neurology.

[24] Sandlers Y., Bobbarala V., Zaman G.S., Desa M.N.M., Akim A.M. (2019). Amino acids profiling for the diagnosis of metabolic disorders. Biochemical Testing-Clinical Correlation and Diagnosis.

[25] Hollak C.E.M., Lachmann R. (2016). Inherited Metabolic Disease in Adults: A Clinical Guide.

[26] Korman S.H., Gutman A. (2002). Pitfalls in the diagnosis of glycine encephalopathy (non-ketotic hyperglycinemia). Dev. Med. Child Neurol..

[27] Canovas R., Cuartero M., Crespo G.A. (2019). Modern creatinine (bio)sensing: Challenges of point-of-care platforms. Biosens. Bioelectron..

[28] Lim M.D., Dickherber A. (2011). Before you analyze a human specimen, think quality, variability, and bias. Anal. Chem..

[29] Yu Z., Kastenmuller G., He Y., Belcredi P., Moller G., Prehn C., Mendes J., Wahl S., Roemisch-Margl W., Ceglarek U. (2011). Differences between human plasma and serum metabolite profiles. PLoS ONE.

[30] Weng N., Jian W. (2017). Targeted Biomarker Quantitation by LC-MS.

[31] Bouatra S., Aziat F., Mandal R., Guo A.C., Wilson M.R., Knox C., Bjorndahl T.C., Krishnamurthy R., Saleem F., Liu P. (2013). The human urine metabolome. PLoS ONE.

[32] Becker K.L. (2001). Principles and Practice of Endocrinology and Metabolism.

[33] Grier R.E., Gahl W.A., Cowan T., Bernardini I., McDowell G.A., Rinaldo P. (2004). Revised sections F7.5 (quantitative amino acid analysis) and F7.6 (qualitative amino acid analysis): American College of Medical Genetics Standards and Guidelines for Clinical Genetics Laboratories, 2003. Genet. Med..

[34] McMillan J.A., Feigin R.D., DeAngelis C., Jones M.D. (2006). Oski’s Pediatrics: Principles & Practice.

[35] Shih V.E. (2003). Amino acid analysis. Physician’s Guide to the Laboratory Diagnosis of Metabolic Diseases.

[36] Van Hove J.L., Curtis Coughlin I., Swanson M., Hennermann J.B. (2019). Nonketotic Hyperglycinemia. GeneReviews^®^ [Internet].

[37] Mandal R., Guo A.C., Chaudhary K.K., Liu P., Yallou F.S., Dong E., Aziat F., Wishart D.S. (2012). Multi-platform characterization of the human cerebrospinal fluid metabolome a comprehensive and quantitative update. Genome Med..

[38] Seo H.-R., Jeong E.-S., Ahmed M.S., Lee H.-K., Jeon S.-W. (2010). Polymeric membrane silver-ion selective electrodes based on schiff base N, *N*′-bis (pyridin-2-ylmethylene) benzene-1,2-diamine. Bull. Korean Chem. Soc..

[39] Dame Z.T., Aziat F., Mandal R., Krishnamurthy R., Bouatra S., Borzouie S., Guo A.C., Sajed T., Deng L., Lin H. (2015). The human saliva metabolome. Metabolomics.

[40] Murphy G.R., Dunstan R.H., Macdonald M.M., Borges N., Radford Z., Sparkes D.L., Dascombe B.J., Roberts T.K. (2019). Relationships between electrolyte and amino acid compositions in sweat during exercise suggest a role for amino acids and K^+^ in reabsorption of Na^+^ and Cl^−^ from sweat. PLoS ONE.

[41] Dunstan R.H., Sparkes D.L., Dascombe B.J., Macdonald M.M., Evans C.A., Stevens C.J., Crompton M.J., Gottfries J., Franks J., Murphy G. (2016). Sweat Facilitated Amino Acid Losses in Male Athletes during Exercise at 32-34 degrees C. PLoS ONE.

[42] Maggs D.G., Jacob R., Rife F., Lange R., Leone P., During M.J., Tamborlane W.V., Sherwin R.S. (1995). Interstitial fluid concentrations of glycerol, glucose, and amino acids in human quadricep muscle and adipose tissue. Evidence for significant lipolysis in skeletal muscle. J. Clin. Investig..

[43] Fernandes J., Saudubray J.-M., Van den Berghe G., Walter J.H. (2006). Inborn Metabolic Diseases: Diagnosis and Treatment.

[44] Garg U., Smith L.D. (2017). Biomarkers in Inborn Errors of Metabolism: Clinical Aspects and Laboratory Determination.

[45] Sharer J.D., De Biase I., Matern D., Young S., Bennett M.J., Tolun A.A., ACMG Laboratory Quality Assurance Committee (2018). Laboratory analysis of amino acids, 2018 revision: A technical standard of the American College of Medical Genetics and Genomics (ACMG). Genet. Med..

[46] Bowron A., Brown A., Deverell D. (2012). Metbionet Guidelines for Amino Acid Analysis.

[47] Tang Y.B., Teng L., Sun F., Wang X.L., Peng L., Cui Y.Y., Hu J.J., Luan X., Zhu L., Chen H.Z. (2012). Determination of glycine in biofluid by hydrophilic interaction chromatography coupled with tandem mass spectrometry and its application to the quantification of glycine released by embryonal carcinoma stem cells. J. Chromatogr. B.

[48] Kugimiya A., Fukada R. (2015). Chemiluminescence detection of serine, proline, glycine, asparagine, leucine, and histidine by rsing corresponding aminoacyl-tRNA synthetases as recognition elements. Appl. Biochem. Biotechnol..

[49] Yoshida H., Kondo K., Yamamoto H., Kageyama N., Ozawa S., Shimbo K., Muramatsu T., Imaizumi A., Mizukoshi T., Masuda J. (2015). Validation of an analytical method for human plasma free amino acids by high-performance liquid chromatography ionization mass spectrometry using automated precolumn derivatization. J. Chromatogr. B.

[50] Moore S., Stein W.H. (1963). Chromatographic determination of amino acids by the use of automatic recording equipment. Methods Enzymol..

[51] Moore S., Spackman D.H., Stein W.H. (1958). Chromatography of amino acids on sulfonated polystyrene resins. An improved system. Anal. Chem..

[52] Thomson A.R., Miles B.J. (1964). Ion-exchange chromatography of amino-acids: Improvements in the single column system. Nature.

[53] Davey J.F., Ersser R.S. (1990). Amino acid analysis of physiological fluids by high-performance liquid chromatography with phenylisothiocyanate derivatization and comparison with ion-exchange chromatography. J. Chromatogr. B.

[54] Li Q.Z., Huang Q.X., Li S.C., Yang M.Z., Rao B. (2012). Simultaneous determination of glutamate, glycine, and alanine in human plasma using precolumn derivatization with 6-aminoquinolyl-*N*-hydroxysuccinimidyl carbamate and high-performance liquid chromatography. Korean J. Physiol. Pharmacol..

[55] Friedman M. (2004). Applications of the ninhydrin reaction for analysis of amino acids, peptides, and proteins to agricultural and biomedical sciences. J. Agric. Food Chem..

[56] Fekkes D. (1996). State-of-the-art of high-performance liquid chromatographic analysis of amino acids in physiological samples. J. Chromatogr. B.

[57] Spackman D.H., Stein W.H., Moore S. (1958). Automatic recording apparatus for use in the chromatography of amino acids. Anal. Chem..

[58] Godel H., Graser T., Földi P., Pfaender P., Fürst P. (1984). Measurement of free amino acids in human biological fluids by high-performance liquid chromatography. J. Chromatogr. A.

[59] Tatsumi M., Hoshino W., Kodama Y., Ueatrongchit T., Takahashi K., Yamaguchi H., Tagami U., Miyano H., Asano Y., Mizukoshi T. (2019). Development of a rapid and simple glycine analysis method using a stable glycine oxidase mutant. Anal. Biochem..

[60] Cooper C., Packer N., Williams K. (2001). Amino Acid Analysis Protocols.

[61] Azadbakht A., Abbasi A. (2014). Fabrication of a high sensitive glycine electrochemical sensor based on immobilization of nanostructured Ni chelidamic acid and bimetallic Au-Pt inorganic-organic hybrid nanocomposite onto glassy carbon modified electrode. Russ. J. Electrochem..

[62] Fan J., Hong J., Hu J.D., Chen J.L. (2012). Ion chromatography based urine amino acid profiling applied for diagnosis of gastric cancer. Gastroenterol. Res. Pract..

[63] Ding Y., Yu H., Mou S. (2002). Direct determination of free amino acids and sugars in green tea by anion-exchange chromatography with integrated pulsed amperometric detection. J. Chromatogr. A.

[64] Bobreshova O.V., Parshina A.V., Safronova E.Y., Titova T.S., Yaroslavtsev A.B. (2015). Potentiometric determination of glycine, alanine, and leucine anions and potassium cations in alkaline solutions using zirconia-modified nafion and MF-4SC membranes. Pet. Chem..

[65] Cuartero M., Parrilla M., Crespo G.A. (2019). Wearable potentiometric sensors for medical applications. Sensors.

[66] Walker V., Mills G. (1995). Quantitative methods for amino acid analysis in biological fluids. Ann. Clin. Biochem..

[67] Heinrikson R.L., Meredith S.C. (1984). Amino acid analysis by reverse-phase high-performance liquid chromatography: Precolumn derivatization with phenylisothiocyanate. Anal. Biochem..

[68] Narayan S.B., Ditewig-Meyers G., Graham K.S., Scott R., Bennett M.J. (2011). Measurement of plasma amino acids by Ultraperformance^®^ Liquid Chromatography. Clin. Chem. Lab. Med..

[69] Sigma-Aldrich: Analytical, Biology, Chemistry & Materials. https://www.sigmaaldrich.com/.

[70] Abcam: Antibodies, Proteins, Kits and Reagents for Life Science. https://www.abcam.com/.

[71] Assay Genie. https://www.assaygenie.com/.

[72] CliniSciences: Reagents and Instruments for Immunology, Cell Biology and Molecular Biology. https://www.clinisciences.com/.

[73] BioVision: Life Science Source Innovation, flexibility, Affordability. https://www.biovision.com/.

[74] Cell Biolabs, Inc.: Creating Solutions for Life Science Research. https://www.cellbiolabs.com/.

[75] Saranya S., Jency Feminus J., Geetha B., Deepa P.N. (2019). Simultaneous detection of glutathione, threonine, and glycine at electrodeposited RuHCF/rGO-modified electrode. Ionics.

[76] Alam M.M., Asiri A.M., Uddin M.T., Islam M.A., Rahman M.M. (2018). In-situ glycine sensor development based ZnO/Al_2_O_3_/Cr_2_O_3_ nanoparticles. ChemistrySelect.

[77] Hasanzadeh M., Sadeghi S., Bageri L., Mokhtarzadeh A., Karimzadeh A., Shadjou N., Mahboob S. (2016). Poly-dopamine-beta-cyclodextrin: A novel nanobiopolymer towards sensing of some amino acids at physiological pH. Mater. Sci. Eng. C Mater. Biol. Appl..

[78] Stadtman E.R. (1993). Oxidation of free amino acids and amino acid residues in proteins by radiolysis and by metal-catalyzed reactions. Annu. Rev. Biochem..

[79] Saghatforoush L., Hasanzadeh M., Shadjou N., Khalilzadeh B. (2011). Deposition of new thia-containing Schiff-base iron (III) complexes onto carbon nanotube-modified glassy carbon electrodes as a biosensor for electrooxidation and determination of amino acids. Electrochim. Acta.

[80] Zheng L., Song J.F. (2009). Nickel(II)-baicalein complex modified multiwall carbon nanotube paste electrode and its electrocatalytic oxidation toward glycine. Anal. Biochem..

[81] Vidotti M., de Torresi S.I.C., Kubota L.T. (2008). Electrochemical oxidation of glycine by doped nickel hydroxide modified electrode. Sens. Actuators B Chem..

[82] Hasanzadeh M., Karim-Nezhad G., Shadjou N., Hajjizadeh M., Khalilzadeh B., Saghatforoush L., Abnosi M.H., Babaei A., Ershad S. (2009). Cobalt hydroxide nanoparticles modified glassy carbon electrode as a biosensor for electrooxidation and determination of some amino acids. Anal. Biochem..

[83] Pérez-Ràfols C., Subirats X., Serrano N., Diaz-Cruz J.M. (2019). New discrimination tools for harvest year and varieties of white wines based on hydrophilic interaction liquid chromatography with amperometric detection. Talanta.

[84] Toyo’oka T. (2009). Recent advances in separation and detection methods for thiol compounds in biological samples. J. Chromatogr. B.

[85] Parshina A.V., Titova T.S., Safronova E.Y., Bobreshova O.V., Yaroslavtsev A.B. (2016). Determination of glycine, alanine, and leucine at different solution pH with the aid of donnan potential sensors based on hybrid membranes. J. Anal. Chem..

[86] Hasanzadeh M., Shadjou N., Omidinia E. (2013). Mesoporous silica (MCM-41)-Fe_2_O_3_ as a novel magnetic nanosensor for determination of trace amounts of amino acids. Colloids Surf. B Biointerfaces.

[87] Roushani M., Shamsipur M., Pourmortazavi S.M. (2012). Amperometric detection of Glycine, l-Serine, and l-Alanine using glassy carbon electrode modified by NiO nanoparticles. J. Appl. Electrochem..

[88] Hasanzadeh M., Shadjou N., Chen S.-T., Sheikhzadeh P. (2012). MCM-41-NH_2_ as an advanced nanocatalyst for electrooxidation and determination of amino acids. Catal. Commun..

[89] St John A., Price C.P. (2014). Existing and emerging technologies for point-of-care testing. Clin. Biochem. Rev..

[90] Pundir C.S., Lata S., Narwal V. (2018). Biosensors for determination of D and L-amino acids: A review. Biosens. Bioelectron..

[91] Dominguez R., Serra B., Reviejo A.J., Pingarron J.M. (2001). Chiral analysis of amino acids using electrochemical composite bienzyme biosensors. Anal. Biochem..

[92] Lee Y.C., Huh M.H. (1999). Development of a biosensor with immobilized l-amino acid oxidase for determination of l-amino acids. J. Food Biochem..

[93] Rathee K., Dhull V., Dhull R., Singh S. (2016). Biosensors based on electrochemical lactate detection: A comprehensive review. Biochem. Biophys. Rep..

[94] Lata S., Pundir C.S. (2013). L-amino acid biosensor based on L-amino acid oxidase immobilized onto NiHCNFe/c-MWCNT/PPy/GC electrode. Int. J. Biol. Macromol..

[95] Zain Z.M., O’Neill R.D., Lowry J.P., Pierce K.W., Tricklebank M., Dewa A., Ab Ghani S. (2010). Development of an implantable D-serine biosensor for in vivo monitoring using mammalian D-amino acid oxidase on a poly (o-phenylenediamine) and Nafion-modified platinum-iridium disk electrode. Biosens. Bioelectron..

[96] Zhang G., Liu D., Shuang S., Martin M.F. (2006). A homocysteine biosensor with eggshell membrane as an enzyme immobilization platform. Sens Actuators B: Chem..

[97] Rosini E., Piubelli L., Molla G., Frattini L., Valentino M., Varriale A., D’Auria S., Pollegioni L. (2014). Novel biosensors based on optimized glycine oxidase. FEBS J..

[98] Job V., Marcone G.L., Pilone M.S., Pollegioni L. (2002). Glycine oxidase from Bacillus subtilis. Characterization of a new flavoprotein. J. Biol. Chem..

[99] Job V., Molla G., Pilone M.S., Pollegioni L. (2002). Overexpression of a recombinant wild-type and His-tagged Bacillus subtilis glycine oxidase in Escherichia coli. Eur. J. Biochem..

[100] Molla G., Motteran L., Job V., Pilone M.S., Pollegioni L. (2003). Kinetic mechanisms of glycine oxidase from Bacillus subtilis. Eur. J. Biochem..

[101] Pedotti M., Rosini E., Molla G., Moschetti T., Savino C., Vallone B., Pollegioni L. (2009). Glyphosate resistance by engineering the flavoenzyme glycine oxidase. J. Biol. Chem..

[102] Zhang W.H., Herde M.K., Mitchell J.A., Whitfield J.H., Wulff A.B., Vongsouthi V., Sanchez-Romero I., Gulakova P.E., Minge D., Breithausen B. (2018). Monitoring hippocampal glycine with the computationally designed optical sensor GlyFS. Nat. Chem. Biol..

